# A new species of *Alopoglossus* lizard (Squamata, Gymnophthalmidae) from the tropical Andes, with a molecular phylogeny of the genus

**DOI:** 10.3897/zookeys.410.7401

**Published:** 2014-05-21

**Authors:** Omar Torres-Carvajal, Simón E. Lobos

**Affiliations:** 1 Escuela de Biología, Pontificia Universidad Católica del Ecuador, Avenida 12 de Octubre y Roca, Apartado 17-01-2184, Quito, Ecuador

**Keywords:** *Alopoglossus*, Andes, Ecuador, Gymnophthalmidae, lizards, systematics

## Abstract

We describe a new species of *Alopoglossus* from the Pacific slopes of the Andes in northern Ecuador based on morphological and molecular evidence. The new species differs most significantly from all other congeners in having a double longitudinal row of widened gular scales, lanceolate dorsal scales in transverse rows, 29–32 dorsal scales in a transverse row at midbody, and 4 longitudinal rows of ventrals at midbody. It is most similar in morphology to *A. festae*, the only species of *Alopoglossus* currently recognized in western Ecuador. We analyze the phylogenetic relationships among species of *Alopoglossus* based on the mitochondrial gene ND4. Cis-Andean [east of the Andes] and Trans-Andean [west of the Andes] species are nested in two separate clades, suggesting that the uplift of these mountains had an important effect in the diversification of *Alopoglossus*. In addition, we present an updated key to the species of *Alopoglossus*.

## Abstract (es)

Describimos una especie nueva de *Alopoglossus* de las estribaciones occidentales de los Andes al norte de Ecuador, en base a evidencia morfológica y molecular. La nueva especie se distingue de otros congenéricos por poseer una hilera longitudinal doble de escamas gulares ensanchadas, escamas dorsales lanceoladas en hileras transversales, 29–32 escamas dorsales sobre una línea transversal al medio cuerpo, y 4 hileras longitudinales de escamas ventrales al medio cuerpo. La nueva especie es semejante en morfología a *A. festae*, la única especie actualmente registrada para el occidente de Ecuador. Analizamos las relaciones filogenéticas entre las especies de *Alopoglossus* en base al gen mitocondrial ND4. Las especies Cis-andinas [este de los Andes] y Trans-andinas [oeste de los Andes] están agrupadas en dos clados distintos, lo cual sugiere que el levantamiento de los Andes tuvo un efecto importante en la diversificación de *Alopoglossus*. Presentamos una clave actualizada para las especies de *Alopoglossus*.

## Introduction

The New World lizard clade Gymnophthalmidae Merrem 1820 includes 241 extant species assigned to 46 taxa traditionally ranked as genera (Uetz 2014). One of them is *Alopoglossus*, which differs from other gymnophthalmid genera except *Ptychoglossus* in having the dorsal surface of the tongue completely covered with anteromedially converging plicae rather than scale-like papillae (Harris 1994; Hoogmoed and Avila-Pires 1992). *Alopoglossus* differs from *Ptychoglossus* (character states for *Ptychoglossus* in parentheses) in having keeled scales on forelimbs (smooth forelimb scales), and rhomboid, laterally imbricating dorsal scales (parallel-sided dorsal scales; Harris 1994).

The close relationship between *Alopoglossus* and *Ptychoglossus* suggested by Harris (1994) based on morphological similarities has been corroborated by phylogenetic analyses of DNA sequence data; these genera are sister taxa and form the clade Alopoglossinae (Castoe et al. 2004). Moreover, this clade seems to be sister to all other gymnophthalmids (Pellegrino et al. 2001; Castoe et al. 2004; Trefaut et al. 2007) as first suggested by Harris (1994). Therefore, studying the phylogenetic systematics of Alopoglossinae is crucial to understand the evolution of gymnophthalmid lizards.

*Alopoglossus* includes six currently recognized species (*Alopoglossus angulatus*, *Alopoglossus atriventris*, *Alopoglossus buckleyi*, *Alopoglossus copii*, *Alopoglossus festae*, and *Alopoglossus lehmanni*) widely distributed across tropical South America (Köhler et al. 2012). Of these, only *Alopoglossus lehmanni* (endemic to Colombia) does not occur in Ecuador; *Alopoglossus festae* occurs west of the Andes, whereas the remaining species occur east of the Andes (Köhler et al. 2012; Torres-Carvajal et al. 2014; Torres-Carvajal 2001). In this paper we describe a new species of *Alopoglossus* from northwestern Ecuador and infer its phylogenetic affinities to other species in the genus as currently understood.

## Materials and methods

### Morphological data

All type specimens of the new species described in this paper are listed in the type series below, and were deposited at the Museo de Zoología, Pontificia Universidad Católica del Ecuador, Quito (QCAZ). Specimens of other species of *Alopoglossus* examined in this study are listed in the Appendix. All measurements were made with digital calipers and recorded to the nearest 0.01 mm: head length (HL), head width (HW), shank length (ShL), axilla-groin distance (AGD), lateral neck scale size (ANS), snout-vent length (SVL), and tail length (TL). Each measurement was taken twice and averaged. Sex was determined by noting the presence of everted hemipenes or by dissection. We follow the terminology of Avila-Pires (1995) and Köhler et al. (2012) for measurements and squamation.

### DNA sequence Data

Total genomic DNA was digested and extracted from liver or muscle tissue using a guanidinium isothiocyanate extraction protocol. Tissue samples were first mixed with Proteinase K and lysis buffer and digested overnight prior to extraction. DNA samples were quantified using a Nanodrop® ND-1000 (NanoDrop Technologies, Inc), re-suspended and diluted to 25 ng/ul in ddH2O prior to amplification.

We amplified 596 nucleotides (nt) of the mitochondrial gene NADH dehydrogenase subunit 4 (ND4) from one individual each of *Alopoglossus angulatus*, *Alopoglossus atriventris*, *Alopoglossus buckleyi*, *Alopoglossus copii*, *Alopoglossus festae*, and the new species described herein. ND4 was amplified using the primers ND4F and ND4R (Pellegrino et al. 2001). Additionally, we used sequences of *Alopoglossus angulatus* (erroneously identified as *Alopoglossus copii* in Pellegrino et al. 2001), *Bachia flavescens*, *Ecpleopus gaudichaudii*, *Heterodactylus imbricatus*, *Ptychoglossus brevifrontalis*, *Rhachisaurus brachylepis* and *Riama unicolor* from GenBank. Gene regions of taxa included in phylogenetic analyses along with their GenBank accession numbers and locality data are shown in Table 1. Amplification of genomic DNA consisted of an initial cycle at 96 °C for 3 min, followed by 40 cycles of a denaturation at 95 °C for 30 s, annealing at 52 °C for 1 min, and extension at 72 °C for 1 min, as well as a final extension at 72 °C for 10 min.

**Table 1.** Vouchers, locality data, and GenBank accession numbers of taxa included in this study. Asterisks indicate new sequences obtained for this study.

Taxon	Voucher	Locality	Genbank number (ND4)	GenSeq nomenclature
*Alopoglossus angulatus* 1^1^	LSUMZ H12692	Ecuador: Sucumbíos: Cuyabeno	AF420865	genseq-4
*Alopoglossus angulatus* 2	QCAZ 8915	Ecuador: Pastaza: Cononaco Lodge	KJ705317	genseq-4
*Alopoglossus atriventris*	QCAZ 5622	Ecuador: Sucumbíos: Cuyabeno	KJ705319	genseq-4
*Alopoglossus buckleyi*	QCAZ 9961	Ecuador: Pastaza: Ingaru Community, Ankaku Reserve	KJ705320	genseq-4
*Alopoglossus copii*	QCAZ 8314	Ecuador: Pastaza: Tarangaro Community, Villano Camp Bloque 10-Agip Oil	KJ705318	genseq-4
*Alopoglossus festae*	QCAZ 9158	Ecuador: Guayas: Bosque Protector Cerro Blanco	KJ705315	genseq-4
*Alopoglossus viridiceps* sp. n.	QCAZ 10670 (holotype)	Ecuador: Pichincha: Nanegal, Santa Lucía Cloud Forest Reserve	KJ705316	genseq-1
*Ptychoglossus brevifrontalis*	MHNSM^2^	—	AY507895	—
*Heterodactylus imbricatus*	LG 1504	Brazil: São Paulo: Serra da Cantareira	AF420885	genseq-4
*Ecpleopus gaudichaudii*	LG 1356	Brazil: São Paulo: Boissucanga	AF420901	genseq-4
*Riama unicolor*	KU 217211	Ecuador: Imbabura: road to Laguna de Mojanda from Tabacundo	AY507893	genseq-4
*Bachia flavescens*	LSUMZ H12977	Brazil: Pará: Agropecuária Treviso, Santarém	AF420869	genseq-4
*Rhachisaurus brachylepis*	MRT 887336	Brazil: Minas Gerais: Serra do Cipó	AF420877	genseq-4

^1^Erroneously identified as *Alopoglossus copii* in Pellegrino et al. (2001).

^2^Voucher number not provided in original publication (Castoe et al. 2004).

### Phylogenetic analyses

Editing, assembly, and alignment of sequences were performed with Geneious ProTM 5.5 (Drummond et al. 2010). Phylogenetic relationships were assessed under a Bayesian approach in MrBayes 3.2.0 (Ronquist and Huelsenbeck 2003). The data matrix was partitioned by codon. The model of character evolution for each partition was obtained in JModeltest (Posada 2008) under the Bayesian information criterion. Four independent analyses were performed to reduce the chance of converging on a local optimum. Each analysis consisted of ten million generations and four Markov chains with default heating values. Trees were sampled every 1,000 generations resulting in 10,000 saved trees per analysis. Stationarity was confirmed by plotting the log-likelihood scores per generation in the program Tracer 1.2 (Rambaut et al. 2013). Additionally, the standard deviation of the partition frequencies and the potential scale reduction factor (Gelman and Rubin 1992) were used as convergence diagnostics for the posterior probabilities of bipartitions and branch lengths, respectively. Adequacy of mixing was assessed by examining the acceptance rates for the parameters in MrBayes and the effective sample sizes (ESS) in Tracer. After analyzing convergence and mixing, 1,000 trees were discarded as “burn-in” from each run. We then confirmed that the four analyses reached stationarity at a similar likelihood score and that the topologies were similar, and used the resultant 36,000 trees to calculate posterior probabilities (PP) for each bipartition on a 50% majority rule consensus tree. Interspecific sequence divergence was assessed with uncorrected distances, which were obtained in PAUP* (Swofford 2003).

## Results

The taxonomic conclusions of this study are based on the observation of morphological features and color patterns, as well as inferred phylogenetic relationships. We consider this information as species delimitation criteria following the general species concept (de Queiroz 1998, 2007).

### Alopoglossus viridiceps sp. n.

http://zoobank.org/1BF11DC5-BD0D-4CF1-ABF4-B5E2884B5812

http://species-id.net/wiki/Alopoglossus_viridiceps

Proposed standard English name: Green-headed shade lizards

Proposed standard Spanish name: Lagartijas de sombra de cabeza verde

#### Holotype.

**Figure 1.**
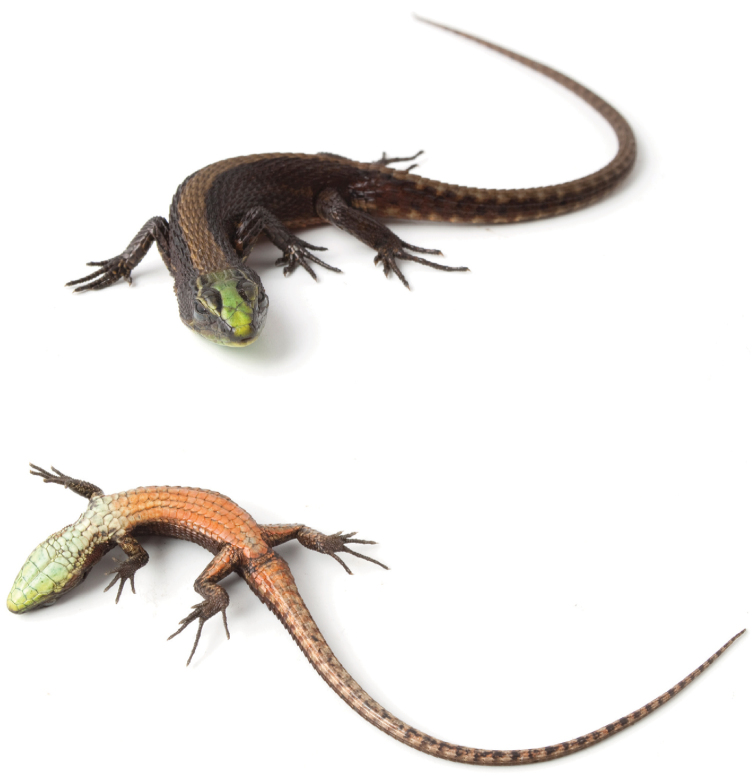
Holotype of *Alopoglossus viridiceps* sp. n. in dorsal (top) and ventral (bottom) views. Male, SVL = 57.89 mm, QCAZ10670. Photographs by OTC.

**Figure 2.**
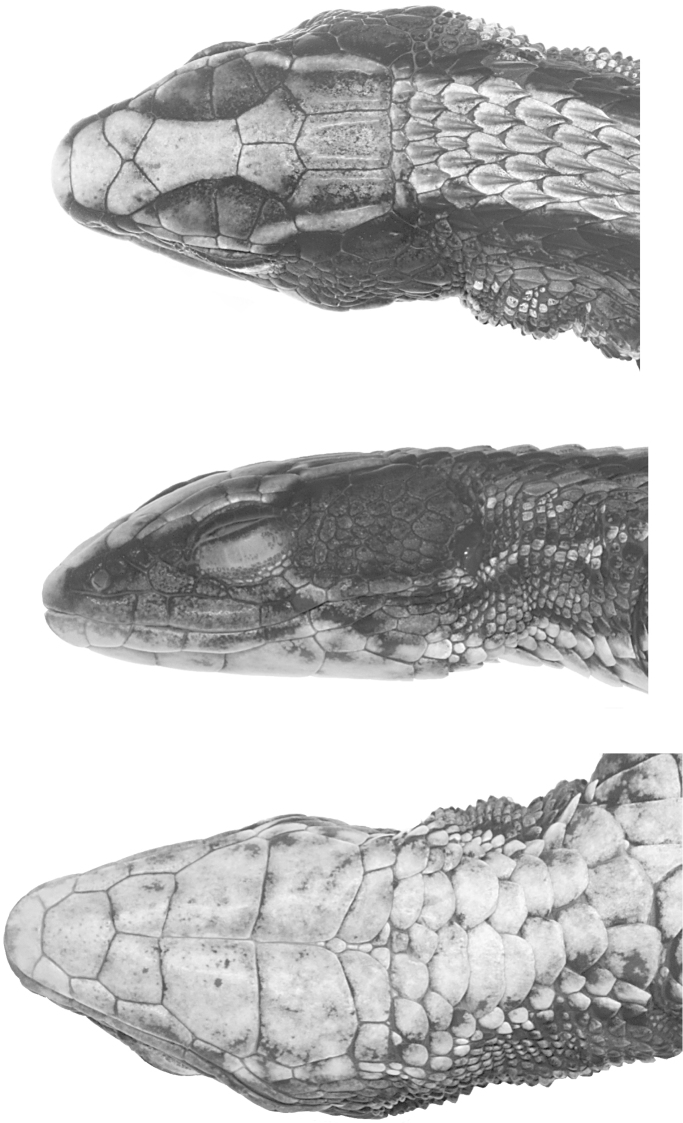
Head of holotype of *Alopoglossus viridiceps* sp. n. (QCAZ10670) in dorsal (top), lateral (middle) and ventral (bottom) views. Photographs by OTC.

QCAZ 10670 (Figs 1, 2), an adult male from Nanegal, Santa Lucia Cloud Forest Reserve, 0.113528°N; -78.6135°W (Decimal Degrees, WGS84), 1742 m, Provincia Pichincha, Ecuador, collected on 27 June 2010 by V. Aguirre-Peñafiel and J. Zanka.

#### Paratypes

**(11).** ECUADOR: Provincia Pichincha: QCAZ 9738, Mindo, Hacienda San Vicente, -0.050720°N, -78.772350°W (DD), 1246 m, collected on 7 August 2009 by S. Poe, E. Schaad, I. Latella, N. Blea, T. Kennedy and F. Ayala-Varela; QCAZ 10821, 10826, Nanegal, Santa Lucía Cloud Forest Reserve, 0.117780°N, -78.607555°W (DD), 1580 m, collected on 9 March 2010 by B. Tolhurst, P. Mafla-Endara, S. Ryan and X. Cueva; QCAZ 11854–55, Nanegal, Santa Lucía Cloud Forest Reserve, trail to waterfalls, 0.109450°N, -78.609380°W (DD), 1645 m, collected on 12 September 2013 by D. Ortiz and O. Torres-Carvajal; QCAZ 11927–29, Nanegal, Santa Lucía Cloud Forest Reserve, 0.113330°N, -78.613280°W (DD), 1736 m, collected on 6 November 2013 by F. Ayala-Varela, E. Carrillo, V. Macias and T. Ostos; QCAZ 10666, 10753, same collection data as holotype, but collected on 14 July 2010 by V. Aguirre-Peñafiel and 26 July 2010 by S. Maddock and V. Aguirre-Peñafiel, respectively. QCAZ 10671, Nanegal, Santa Lucía Cloud Forest Reserve, 0.119280°N, -78.596470°W (DD), 1911 m, collected on 29 June 2010 by M.A. Chinchero.

#### Diagnosis.

**Figure 3.**
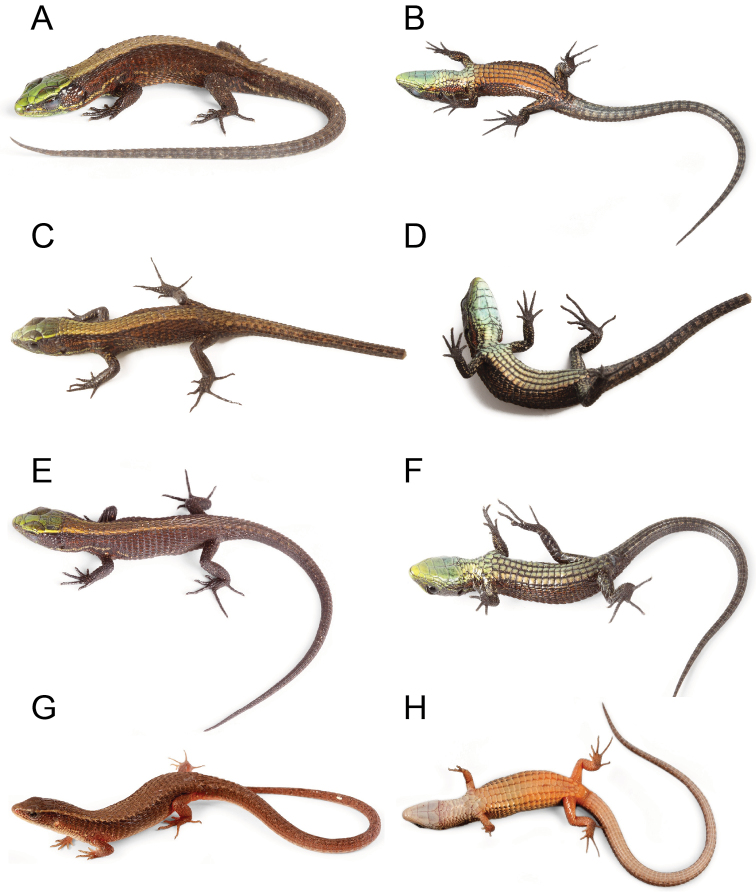
Species of *Alopoglossus* from western Ecuador. **A, B** *Alopoglossus viridiceps* sp. n., paratype QCAZ11854, juvenile male, SVL = 38.67 mm **C, D** *Alopoglossus viridiceps* sp. n., paratype QCAZ10671, juvenile female, SVL = 33.80 mm **E, F** *Alopoglossus viridiceps* sp. n., paratype QCAZ11855, juvenile, SVL = 31.59 mm **G, H** *Alopoglossus festae*, QCAZ 9161, female, SVL = 46.89 mm.

*Alopoglossus viridiceps* can be distinguished from all other known congeners except *Alopoglossus festae* by having a double longitudinal row of widened gular scales and lanceolate dorsal scales in transverse rows. From *Alopoglossus festae* (character states in parentheses, taken from Köhler et al. 2012), the new species differs in having 29–32 dorsal scales in a transverse row at midbody (16–24, mean = 19.14 ± 2.25), four ventral scales in a transverse row at midbody (six), and a distinct longitudinal light stripe from mouth commissure to shoulder (Fig. 3). Scale counts and measurements of *Alopoglossus festae* and *Alopoglossus viridiceps* are presented in Table 2.

**Table 2.** Scale counts and measurements of *Alopoglossus festae* and *Alopoglossus viridiceps*. Range (first line) and mean ± SD (second line) are presented when appropriate. Data for *Alopoglossus festae* was taken from Köhler et al. (2012). Sample size for *Alopoglossus viridiceps* is presented in parentheses if different from that in heading.

Character	*Alopoglossus festae* Köhler et al. (2012)	*Alopoglossus viridiceps* sp. n. *N* = 12
Maximum SVL (snout—vent length) males	60.0 mm	64.13 mm
Maximum SVL females	64.5 mm	57.22 mm
Longitudinal dorsal count	29–31 30.14 ± 0.64	30–33 31.33 ± 0.26
Transversal dorsal count	16–24 19.14 ± 2.25	29–32 30.33 ± 0.26
Longitudinal ventral count	16–19 17.29 ± 1.03	17–18 (10) 17.2 ± 0.12
Transversal ventral count	6 6.00 ± 0.00	4 4.00 ± 0.00
Gulars rows	6–8 7.25 ± 0.68	7–8 7.08 ± 0.08
Frontonasals	1 1.00 ± 0.00	1 1.00 ± 0.00
Supraoculars	3–4 3.97 ± 0.18	4 4.00 ± 0.00
Anterior supralabials	3 3.00 ± 0.00	3 3.00 ± 0.00
Posterior supralabials	3–4 3.95 ± 0.21	2 2.00 ± 0.00
Infralabials	4–5 4.82 ± 0.38	4 4.00 ± 0.00
Scales between third chin shields	1–2 1.08 ± 0.26	1 1.00 ± 0.00
Transparent eye disk fragments	4–6 4.90 ± 0.64	6–8 6.6 ± 0.19
Lamellae fourth toe	17–24 18.77 ± 1.52	15–17 16.17 ± 0.21
Femoral pores	3–8 5.67 ± 1.15	1 (10) 1.00 ± 0.00
Tail length / SVL (%)	134.1–222.5 183.66 ± 22.54	164.56–199.92 (5) 177.83 ± 5.56
Head length / SVL (%)	20.4–25.5 22.73 ± 1.36	22.78–27.78 25.55 ± 0.41
Head width / SVL (%)	13.5–19.3 15.90 ± 1.34	15.85–19.99 18.11 ± 0.34
Shank length / SVL (%)	13.0–18.1 15.66 ± 1.22	15.81–19.02 17.88 ± 0.31
Axilla-groin distance / SVL (%)	37.5–50.0 44.23 ± 2.90	40.68–49.25 45.30 ± 0.83
Lateral neck scale size / head length (%)	1.3–5.5 3.08 ± 0.95	2.41–4.68 3.25 ± 0.16

#### Description of holotype.

Male (Figs 1, 2); SVL= 57.89; TL/SVL= 1.99; HL/SVL = 0.24; HW/SVL = 0.16; ShL/SVL = 0.16; AGD/SVL = 0.43; ANS/HL = 3.58.

Rostral hexagonal, 2.08 times as wide as high, visible from above, in broad contact with frontonasal. Frontonasal irregularly pentagonal, wider than long, laterally in contact with nasal. Prefrontals irregularly pentagonal, nearly as wide as long, with medial suture; laterally in contact with nasal, loreal, and first and second supraocular. Frontal irregularly hexagonal, nearly twice as long as wide, slightly wider anteriorly; at each side in contact with supraoculars II–III. Frontoparietals irregularly pentagonal, longer than wide, with a wide medial suture; each in contact with supraoculars III–IV. Interparietal pentagonal, lateral borders parallel to each other. A pair of irregularly hexagonal parietals, approximately as wide and as long as interparietal. Interparietal and parietals forming slightly undulating posterior head margin. Occipitals absent. Four supraoculars, first one smallest and second one largest. Four elongate superciliaries, first one widest, followed by a postsuperciliary scale, which is also in contact with supraocular IV and anterior supratemporal. Nasal divided, irregularly pentagonal, longer than wide, in contact with rostral anteriorly, first and second supralabials ventrally, frontonasal and prefrontals dorsally, loreal posterodorsally, and frenocular posteroventrally. Nostril in lower part of nasal, directed lateroposteriorly. Loreal small, quadrangular. Frenocular in contact with nasal, separating loreal from supralabials. Three suboculars, the one below eye very elongated (nearly three times the size of adjacent suboculars). Posterior subocular continuous with three postoculars. Semitransparent disc in lower eyelid with vertical sections delimiting six large scales on right side and five scales on left side. Five supralabials, third one longest and below center of eye. Two postsupralabials. Temporals small, irregularly polygonal, juxtaposed, keeled. Two large supratemporal scales, posterior one keeled. Ear opening vertically oval, without denticulate margins. Tympanum recessed into a short auditory meatus. All dorsal and lateral head scales juxtaposed. Interparietal and parietals with lateral ridges, other dorsal head scales smooth. Mental trapezoidal, anterior margin nearly forming a semicircle. Postmental irregularly heptagonal, wider than long. Four infralabials, third one longest and below center of eye. One postinfralabial. Three pairs of chin shields, first two in contact medially and with infralabials; third one in contact medially but separated from infralabials. Third pair of chin shields separated from gulars by two transverse rows of scales. Anterior row composed laterally by two scales (one on each side) similar in size to the scales on the posterior row, and medially by two enlarged scales (not in contact medially) similar in size to the enlarged gulars. Gulars imbricate, smooth, in four longitudinal rows, the medial double row formed by five pairs of distinctly widened scales. Posterior row (collar) with five scales, the medial three distinctly widened (Fig. 2).

Scales on nape similar to dorsals, except that anterior ones are shorter. Scales on sides of neck small, keeled and mostly granular. Dorsals and scales on flanks lanceolate, strongly keeled and mucronate, imbricate, in transverse rows; number of scales along a middorsal line from nape to base of tail 30; transversal dorsal count 31. Ventrals smooth, imbricate, with round posterior margin; 18 in a longitudinal count; four in a transverse count. Scales on flanks similar to dorsals. One femoral pore on each side, in preanal position, separated from each other by four ventral scales. Scales on tail keeled, slightly mucronate, imbricate; in transverse and longitudinal rows; dorsal keels sharp, forming four distinct longitudinal ridges. Scales on limbs mostly rhomboidal, imbricate, sharply keeled, and mucronate; smooth on ventral aspect of hind limbs, small and keeled or tuberculate on ventral aspect of upper arms and posterior aspect of thighs. Subdigital lamellae of fingers and toes single, transversely enlarged and smooth; 20 under fourth toe.

#### Color in life of holotype

(Fig. 1). Dorsal background uniformly dark brown with a wide light brown vertebral stripe extending from occiput onto tail; vertebral stripe wider anteriorly; dorsal surface of head bright metallic green medially (rostral, frontonasal, prefrontals, frontal and frontoparietals) and dark brown laterally (supraoculars and supratemporals), with a lateral bright green stripe on each side extending posteriorly from the border between the loreal and the first supraocular, over the superciliaries, to the lateral border of the parietal; lateral aspect of neck with a longitudinal yellowish-green stripe extending posteriorly from mouth commissure, over ventral margin of tympanum, to shoulder; most scales between lateral neck stripe and gular region reddish brown forming a short irregular stripe between last infralabial and shoulder; ventral surface of head light green, brighter laterally; gular and pectoral regions same tone as chin shields but lighter; ventral aspect of body orange with scattered light green or light blue small marks; ventral aspect of tail with dark brown marks that form transverse bars on the posterior half.

#### Variation.

Intraspecific variation in scale counts and measurements in *Alopoglossus viridiceps* sp. n. is presented in Table 2. Color in preservative of holotype is similar to its color in life, except that the bright green tones of the head and orange tones of the venter have faded away.

Color in life of juvenile paratypes QCAZ10671, QCAZ11854–55 is similar to that of the holotype except that these juveniles have a reddish-brown longitudinal stripe extending from the dorsal border of the tympanum to the shoulder and fading away on the flanks (Fig. 3). The orange ventral coloration of male juvenile QCAZ11854 does not extend onto tail as in the holotype; female juvenile QCAZ10671 and juvenile QCAZ11855 (undetermined sex) have a light yellowish green background color on the venter, similar to that on gular region and chin (Fig. 3).

#### Distribution and ecology.

**Figure 4.**
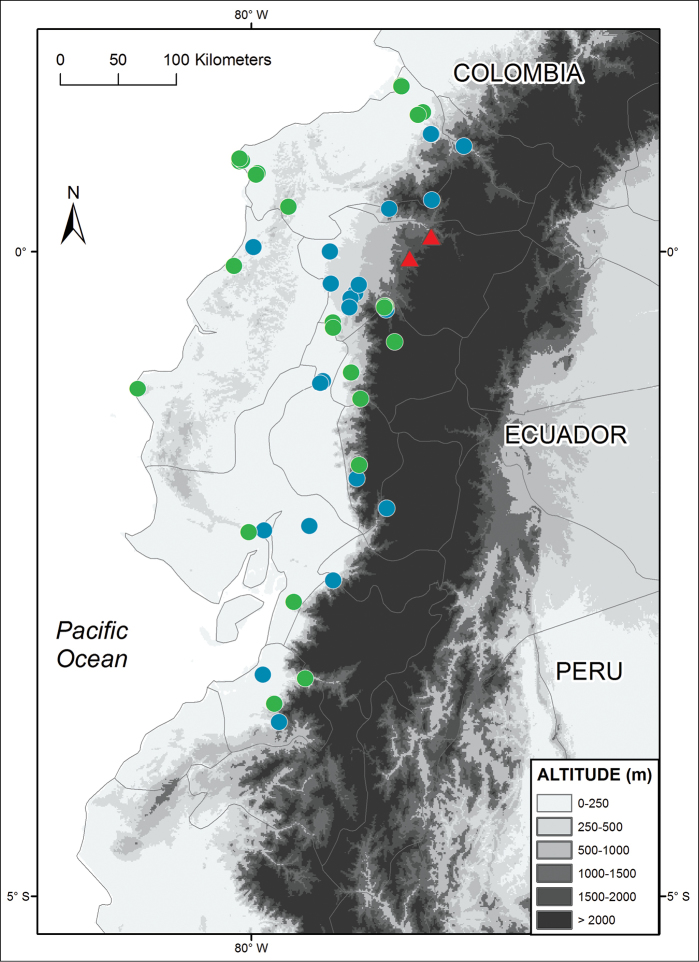
Distribution of *Alopoglossus viridiceps* sp. n. (triangles) and its sister species *Alopoglossus festae* (circles) in Ecuador. Locality data for *Alopoglossus festae* was taken both from the literature (blue circles; Almendáriz and Carr 2012; Köhler et al. 2012) and museum specimens (green circles; see Appendix).

*Alopoglossus viridiceps* sp. n. inhabits cloud forests on the Pacific slopes of the Andes in northwestern Ecuador (Fig. 4). It occurs at elevations of 1246–1911 m in the province of Pichincha. Most type specimens were collected at Santa Lucía Cloud Forest Reserve, which extends between 1400–2560 m and has an area of 756 ha; annual precipitation ranges from 1500 to 2800 mm, and average annual temperature is recorded at 16 °C (Rivas-Martínez and Navarro 1995). Specimens of *Alopoglossus viridiceps* sp. n. were found active between 9h30–11h30 on leaf litter in primary forest, or on the border of sugar cane plantations. Other species of small ground lizards collected in the same area include the sphaerodactylid gecko *Lepidoblepharis conolepis*, the gymnophthalmids *Cercosaura vertebralis* and *Echinosaura brachycephala*, as well as an undescribed species of the gymnophthalmid genus *Riama*.

#### Etymology.

The specific epithet *viridiceps* is an adjective derived from the Latin words “viridis” and “ceps”, which mean “green” and “head”, respectively. It refers to the distinctive bright green coloration of the dorsal and ventral aspects of the head of *Alopoglossus viridiceps* sp. n.

#### Phylogenetic relationships.

**Figure 5.**
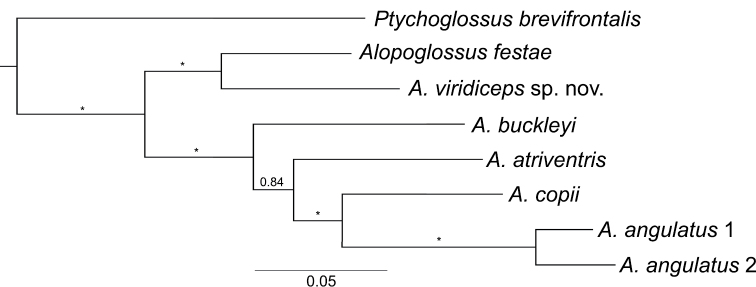
Phylogeny of Alopoglossinae. Majority rule (50%) consensus tree of 36,000 trees obtained from a Bayesian analysis of the mitochondrial gene ND4 and 8 specimens. Asterisks correspond to posterior probability values ≥ 0.97. Voucher information is presented in Table 1.

Of the 596 nucleotide characters included in our analysis 290 were constant, 70 parsimony uninformative, and 236 were parsimony informative. Selected models of evolution were 012013+I+G+F, TPM2uf+I+G, and 010220+I+G+F for ND4 partitions codon 1, 2, and 3, respectively. The resulting 50% majority rule consensus tree (Fig. 5) supports strongly (PP=1) the monophyly of Alopoglossinae (i.e., *Ptychoglossus* and *Alopoglossus*) and *Alopoglossus*. Within *Alopoglossus* there is a basal split into two strongly supported (PP=1) clades, one containing trans-Andean taxa (*Alopoglossus festae* and *Alopoglossus viridiceps* sp. n.), and the other including cis-Andean taxa (*Alopoglossus angulatus*, *Alopoglossus atriventris*, *Alopoglossus buckleyi*, and *Alopoglossus copii*). Within the cis-Andean clade, *Alopoglossus angulatus* and *Alopoglossus copii* are recovered as sister species with maximum support (PP=1), forming a clade sister to *Alopoglossus atriventris* (PP=0.84); *Alopoglossus buckleyi* is sister to all other cis-Andean species (Fig. 5).

Uncorrected genetic distances for ND4 are presented in Table 3. Distance values between *Ptychoglossus brevifrontalis* and species of *Alopoglossus* ranged between 0.197–0.225. The genetic distance between *Alopoglossus viridiceps* sp. n. and its sister species *Alopoglossus festae* (0.124) is slightly lower than all other interspecific distance values within *Alopoglossus* (0.148–0.185). *Alopoglossus angulatus*, the only species for which we had two samples, had an intraspecific distance value of 0.06.

**Table 3.** Pairwise ND4 genetic distances (uncorrected) among samples of alopoglossines included in this study.

Taxon	*Alopoglossus angulatus* 1	*Alopoglossus angulatus* 2	*Alopoglossus atriventris*	*Alopoglossus buckleyi*	*Alopoglossus copii*	*Alopoglossus festae*	*Alopoglossus viridiceps*
*Alopoglossus angulatus* 1							
*Alopoglossus angulatus* 2	0.060						
*Alopoglossus atriventris*	0.159	0.158					
*Alopoglossus buckleyi*	0.171	0.166	0.161				
*Alopoglossus copii*	0.149	0.156	0.148	0.158			
*Alopoglossus festae*	0.163	0.169	0.158	0.171	0.178		
*Alopoglossus viridiceps*	0.185	0.185	0.178	0.168	0.180	0.124	
*Ptychoglossus brevifrontalis*	0.208	0.213	0.205	0.219	0.225	0.197	0.205

## Discussion

The phylogenetic tree presented in this paper (Fig. 5) supports strongly the monophyly of *Alopoglossus* and its sister taxon relationship with *Ptychoglossus*, as suggested by previous authors based on morphological evidence (Harris 1994). The basal split between cis-Andean and trans-Andean species of *Alopoglossus* suggests that the uplift of the Andes represented an important event that allowed allopatric speciation in *Alopoglossus*, whether resulting from dispersal or vicariance.

*Alopoglossus viridiceps* sp. n. can be distinguished readily from its sister species *Alopoglossus festae* based on morphological and color characters (see Diagnosis). *Alopoglossus festae* occurs as close as 30 km W from the known distribution of *Alopoglossus viridiceps* sp. n. below 1000 m (Fig. 4), suggesting that these species originated by allopatric or parapatric speciation. Although we did not attempt to examine variation within *Alopoglossus festae*, we found differences among some populations of this species that suggest it might represent a species complex as currently circumscribed. A more detailed systematic study of *Alopoglossus festae*, as well as all species of *Alopoglossus* east of the Andes in Ecuador with extensive geographic sampling is underway.

*Alopoglossus viridiceps* sp. n. is one of two new species of lizards that have been discovered recently in the same area. The other one is an undescribed species of the gymnophthalmid genus *Riama*. These discoveries indicate that the herpetofauna of the cloud forests in this region is more diverse in species numbers than previously thought. We recommend increasing field surveys in this region as it includes several protected areas (e.g., Santa Lucía Cloud Forest Reserve, Mindo-Nambillo Protected forest, Maquipucuna Reserve, El Cedral Ecolodge) that provide an opportunity to find species of lizards that might not occur elsewhere.

### Key to the species of *Alopoglossus* (modified from Köhler et al. 2012)

**Table:** 

1	A double longitudinal row of widened gular scales; dorsal scales lanceolate in transverse rows only	2
–	No double longitudinal row of widened gular scales; dorsal scales hexagonal in transverse rows only or rhomboidal in oblique and transverse rows	3
2	Fewer than 25 dorsal scales in a transverse row at midbody; no distinct light stripe from mouth commissure to shoulder	*Alopoglossus festae*
–	More than 29 dorsal scales in a transverse row at midbody; distinct light stripe from mouth commissure to shoulder	*Alopoglossus viridiceps*
3	Dorsal scales hexagonal with parallel lateral edges, in transverse rows only; transverse ventral count 10	*Alopoglossus lehmanni*
–	Dorsal scales rhomboidal or lanceolate, in oblique and transverse rows; transverse ventral count 4–8	4
4	Keels on posterior part of dorsum form longitudinal ridges; scales on side of neck large and conical with apparent bare skin between conical scales; longitudinal dorsal count 23–24	*Alopoglossus copii*
–	Keels on posterior part of dorsum do not form longitudinal ridges; scales on side of neck small and granular or keeled and somewhat imbricate without apparent bare skin between scales; longitudinal dorsal count 24–34	5
5	Scales on side of neck leaf-like (similar in shape to dorsal scales, nongranular) and somewhat imbricate; longitudinal dorsal count 24–28	*Alopoglossus angulatus*
–	Scales on side of neck small and granular; longitudinal dorsal count 29–34	6
6	Ventral scales smooth	*Alopoglossus buckleyi*
–	Ventral scales distinctly keeled	*Alopoglossus atriventris*

## Supplementary Material

XML Treatment for Alopoglossus viridiceps

## Appendix

**Specimens examined**

*Alopoglossus festae* – ECUADOR: *Azuay*: QCAZ 5002, Recinto La López, campamento minero Produmin S.A., -3.08732°N, -79.71493°W, 425 m; QCAZ 7249, Sarayunga 41 km desde Santa Isabel por la vía hacia Pasaje, -3.31423°N, -79.58145°W; *Bolivar*: QCAZ 7952-53, Recinto San Francisco, -1.65977°N, -79.16384°W, 1379 m; *Cotopaxi*: QCAZ 7250, between Corazón and Moraspungo, -1.14643°N, -79.15170°W, 873 m; QCAZ 2764, 2783, La Maná, -0.93839°N, -79.22536°W, 250 m; QCAZ 10386, Naranjito, Bosque Integral Otonga (BIO), Finca Familia Tapia, -0.42322°N, -78.95702°W, 1425 m; QCAZ 7835, San Francisco de Las Pampas, -0.43326°N, -78.966733°W; QCAZ 5308, Sigchos, -0.42371°N, -78.96765°W, 1500 m; QCAZ 3527, Sigchos, Asache, -0.70124°N, -78.88795°W; *El Oro*, QCAZ 8987, 8991, Bella María, near Valle Hermoso, -3.51168°N, -79.82026837°W; *Esmeraldas*: QCAZ 6400, 7km W Durango, 1.07961°N, -78.66817°W, 220m; QCAZ 5740, 5742, surroundings of Caimito, río Tongora headwaters, 0.70425°N, -80.07254°W, 14 m; QCAZ 5261, Caimito, 0.69755°N, -80.09014°W, 121 m; QCAZ 8442-43, Caimito, 0.72096°N, -80.09117°W, 518 m; QCAZ 6990, El Barro, 0.59500°N, -79.96300°W; QCAZ 6190, La Tortuga, 0.60792°N, -79.95050°W, 91 m; QCAZ 7554, 5 km from Playón de San Francisco on way to Urbina, 1.05983°N, -78.70742°W, 136 m; QCAZ 6874, Bilsa Ecological Reserve, 0.34705°N, -79.71140°W; QCAZ 1404, San Lorenzo, 1.28130°N, -78.83476°W; *Guayas*: QCAZ 9114, 9121, 9131, 9147, 9158, 9161, Bosque Protector Cerro Blanco, -2.17833°N, -80.02139°W, 281 m; QCAZ 3325-26, Naranjal, 15 min on road to Pasaje, Nueva Unión Campesina cooperative, -2.72159°N, -79.67007°W, 45 m; *Los Ríos*: QCAZ 2180, 2222, Centro Científico Río Palenque (CCRP), 48 km on road Santo Domingo-Quevedo, -0.592133°N, -79.36395°W; *Manabí*: QCAZ 11428, Jama Coaque, 3.12 km W Camarones, -0.1187°N, -80.1231°W, 349 m; QCAZ 11508, 11514, Pacoche Lodge, -1.06675°N, -80.88100°W, 335 m; QCAZ 4643, 5885, Jama Coaque Reserve, -0.09098°N, -80.14719°W; *Pichincha*, QCAZ 5080, río Chirapi headwaters, 0.116°N, -78.91°W; QCAZ 1425, ENDESA, forest reserve near Puerto Quito, 0.098°N, -79.117°W; QCAZ 6735, Mindo, -0.05067°N, -78.77188°W.
